# Alumni Association, Boston Dental College

**Published:** 1899-05

**Authors:** Marion L. Woodward

**Affiliations:** Secretary Alumni Association, Boston Dental College


### ALUMNI ASSOCIATION, BOSTON DENTAL COLLEGE.

A MEETING of the Alumni Association of the Boston Dental College was held February 8, 1899. At the conclusion of the routine business the President called upon the essayist of the evening, Dr. E. C. Kirk, of Philadelphia, to read his paper on "The Predisposing Factor in Dental Disorders."

(For Dr. Kirk's paper, see page 277.)

### DISCUSSION.

*Dr. H. H. Piper.*—So far as I am concerned, I think that I would have preferred it if the lights had been kept down, and I could have gone from this room to my home and meditated on the lecture that we have heard. It is a very great pleasure to have the subject presented so ably.

Although we are restricted in our observations, as dentists, to one part of the human body, it is evident that we are apt to scatter in what we have to say regarding our professional work.

It is a great grief to me that I have not more time to give to that broad foundation in dental education which I think every dentist ought to possess,—namely, a knowledge of medicine and of the body as a whole; so that in an examination of the mouth one might the more intelligently pass judgment on what he sees. I almost envy those dentists who, fifty years ago, attended the Harvard Medical School and then went into a dental office and elected to practise dentistry. We all feel that it would be a great advantage to us if we could only have some of that broader knowledge. But it is too late for most of us. It can only be obtained by years of patient study, but it is a satisfaction to think that the students of the future will have this foundation, and will be able to carry on investigations better than we. It will be impossible for me, therefore, to follow, in a suitable way, the points of this lecture; but there have been some responsive chords struck, and one of them is that we want to know more of the relation between dentistry and medicine: the dentist should know more of medicine, and the physician more of dentistry, and there should be more reciprocal action.

The lecturer touched on the matter of nutrition, which I believe will be one of the important questions in the future. We, as dentists, pay very little attention to the human body outside of the mouth; we keep pretty closely to our field of operation, and our thought is largely taken up with the best thing to be done under the circumstances, preventing the progress of disease, in keeping the mouth as healthy as possible; but the whole realm of the body, outside of the mouth, the circulation of the blood, the question of nutrition, and so forth, we are apt to ignore. Surely, as Americans, this question appeals to us more and more every year, and it becomes more and more apparent that the conditions of American life are responsible for the condition of American teeth. More or less, as dentists, we have observed that the mouth is connected with the whole body and subjected to the conditions that govern the whole body. I do not forget the fact that the enamel of the teeth is practically outside the realm of nutrition; but, otherwise than that, nutrition affects profoundly all the portions of the mouth, and we cannot, if we do our full duty, neglect those marvellously interesting problems which arise in this connection. Our high-school children are sent to school half-past eight o'clock in the morning, and they are obliged to take the first meal of the day before that time; they are supposed to take no other meal until two o'clock in the afternoon. How the child is fed in the interim is not determined by the parent, but by the child as he sees fit. Is it a wonder that the digestion of the child becomes impaired? Every one of us has been called on to attend children who have suffered from these conditions of American life.

To illustrate how closely related the solid structure of the teeth is to the matter of nutrition, I might say that I have had young patients come with teeth in perfect condition, and I have known them to remain in that condition during their first or second year in the high school, when suddenly a dozen cavities would appear, clearly connected with impaired digestion. This will, at least, illustrate forcibly the influence of malnutrition in some instances. There are, apparently, striking exceptions, as in the case of those persons who have the strongest, densest, and healthiest teeth in spite of disease and invalidism, just as there are persons in perfect health whose teeth are in the worst condition.

I was interested in the way that the lecturer held to the point that phagedenic pericementitis is a symptom of a disease rather than a disease.

You are aware, of course, that we know nothing of dentistry beyond a hundred years ago, and that all the conditions which governed the teeth of our ancestors are, more or less, wrapped in obscurity; but now and then there is a little light let in on the subject. Caries is not found to any great extent in teeth of skulls which are known to be very old; in the teeth of those peoples which did not eat cooked starches. Three hundred years ago, under Queen Elizabeth, when these were freely used, caries was increasing. We know pretty well what the conditions of life at that time were: the men were wine drinkers; that has something to do with the gout. So we are brought in touch with the paper of the evening. There is one other interesting fact in connection with the English people, and that is that they were not subject to the nervous disorders to which we are to-day, they did not suffer from dyspepsia, and their teeth, though not perfect, were less carious than ours. I believe that it is with us a good deal a question of digestion, of nutrition. It seems to me rather interesting that in the works of the Elizabethan dramatists, we find less evidence of caries than of phagedenic pericementitis.

I must beg your pardon for taking up so much of your time.

*Dr. D. M. Clapp.*—It seems to me that it is a very great thing that the dentists of Boston could have presented to them for the first time the paper and illustrations that we have seen to-night. In all my attempts to benefit my patients I never feel quite so helpless as when a case of this kind presents itself. I palliate the trouble, and sometimes the patient gets over it; whether I have anything to do with the recovery I cannot say. But, certainly, while I have little to say on the subject, I can say this, that I fully appreciate the endeavors of others to bring light upon it. It is only by great application, long study, experiment, and observation that we shall ever become able to cope with this pathological condition. Consequently, I bow to men who put themselves into harness and work that the public may be benefited by it, and I wish personally to give my thanks to those men who are doing so much for the profession and, through the profession, for the people at large.

*Dr. R. R. Andrews.*—The loop-like formations shown on the screen to-night are more or less familiar to all who have studied the microscopical structure of the pericementum. I have heretofore regarded them as capillary loops, filled with blood-corpuscles. Those shown by the highest powers—about twelve hundred diameters—look very like blood-corpuscles filling the capillaries; still I have a great deal of faith in what Dr. Black may say. I shall investigate these appearances with a new interest. It is only within a few years that we have been able to demonstrate the appearance of tissues in this way. The slides are beautiful and show the tissue perfectly. I have very little to add to what has already been said. I have not thought there were glandular formations in the pericementum of the human tooth; I have often noticed these appearances but have never given them any attention, thinking them merely capillary loops. In the treatment of the cases spoken of by the essayist I have had my failures and my successes, and I have no doubt that a gouty diathesis has a good deal to do with the trouble. I have been much interested in the way some of the tartar formations found on the roots of some teeth are formed. I have a fine specimen that shows three nodular formations which are located near the apex of the root that look as if they had been formed by a vessel of the pericementum, which had gradually given out lime-salts, and these, blending with the organic fluids, had gradually formed these nodular bodies. Whether this is so I cannot positively say. Professor Kirk, in his paper, has taken a different course from what I was led to expect by the printed card. I supposed we were to hear something of the vital principle of the organic structure of the tooth itself. Observation has taught me that there is a vital power within this organic structure that will, in the healthy patient, resist in a measure the inroads of organisms which produce decay. In very many cases the germs of caries enter the tooth-structure in a minute pit or fissure, and follow the canals of the dentine until they find resistance by a vital principle from the pulp. They then spread in the direction of the weakest part, the approximal surfaces. Many a tiny pit in the enamel entered by the drill finds a large cavity just within and perhaps exposing an approximal surface of decay, yet having a considerable amount of normal dentine between the infected portion and the pulp.

In our colleges students that are overworked lose their health, teeth are found to be decaying rapidly, and they are sent away for a brief rest and a change of scene. This is usually all that is needed to bring back the normal condition. On the subject of the paper as presented, I can only express to the essayist the pleasure I have had in hearing it. In the future I may be able to say something more about the appearances which Dr. Black calls pericemental glands, and which I have always supposed were the loopings of the blood-vessels in this tissue.

*Dr. E. H. Smith.*—I thought that I had so arranged with Professor Andrews that I would not be called upon to-night. I will frankly say, gentlemen, that I cannot give any light on the subject that Professor Kirk has so ably presented. It has certainly been a very great pleasure to me to be present this evening, and I feel under obligation for so vivid a presentation of a subject that is always interesting.

*Dr. B. H. Strout.*—It is some years ago, I do not know just how many, since the first paper was published connecting the uric acid diathesis with pyorrhœa alveolaris. Since that time I have tried to find some connection between the disease and certain conditions (such as gout, rheumatism, etc.) in practical experience, and so far (perhaps I do not know what phagedenic pericementitis is) have absolutely failed to find any relationship with any particular diathesis. There has been, in many cases, so marked a disparity that I have not been able to connect the two conditions. I do not like to seem in opposition to so high an authority, but I cannot make it clear to myself that these two things are cause and effect. A great many of the patients whom I see afflicted in this way are robust and healthy people; you inquire about rheumatic troubles and they never heard of them; some have, but the majority have not. I have in mind a particular patient of mine, a man who is a school-teacher; he takes a great deal of exercise, he is healthy in every way, except that he is laid up every winter with rheumatism. His teeth are the best I have ever seen for a man of his age, with never any trouble from disease of the peridental membrane. Another is a physician of large practice; his teeth are also perfect in number and position, but it is a constant struggle from year to year to keep those teeth from loosening. He suffers also from marked erosion. He is a man who knows what the uric acid diathesis is, but we cannot find any connection between the two in this case.

*Dr. I. J. Wetherby.*—Mr. President, I think that the subject has been so much exhausted by the essayist of the evening that there is nothing for me to say. As to trouble growing out of uric acid in connection with the teeth, I believe that it is absolutely true, and I have long been persuaded that Professor Peirce's view was the correct one. I made the matter one of considerable study in the past, and I am firm in my views. There is one question that I wish to ask Professor Kirk concerning the peridental membrane, and that is, whether there are two membranes or one. That is, when you extract a tooth, do you leave the socket covered with a membrane, and is the root itself covered by a membrane?

*Professor Kirk.*—It may or it may not be. It is a membrane with a double function.

*Dr. Wetherby.*—That is what I believed. It has been denied by some, but if we examine the socket we find the walls covered by a membrane.

*Dr. Woodward.*—What regimen should be adopted to bring a patient back to normal health?

*Dr. Kirk.*—It simply means carefully studying all the conditions and adopting such measures as will tend to bring about a return of health. You will generally find that these people are bad eaters; that they have picked out a diet according to their palates. It means correction of the errors of living, correcting the habit of "bolting" food or the habit of eating too rapidly or too much. We often find that these people drink very little water. I ask a patient, "What did you have for breakfast this morning? for dinner? for lunch? Do you take much tea? coffee?" Another thing is the quantity of food. Up to a certain age the appetite of a child is very large, he is then building up the tissues, and the appetite is greatly in excess of what would be normal in the adult, and the faulty habit of eating in excess is sometimes formed. I say to the patient, "You would not fill up the stove with coal and close up the draughts; the fire would go out. Proportion the fuel and draught so as to obtain the largest amount of energy and be sure to get your fire well raked, and not let it fill up with ashes." That is the general idea.

*Dr. S. G. Stevens.*—Is there any relation between the pulp and this pericementitis? We have in our city an eminent dentist, who is also an M.D., who claims that there is a very vital connection between the pulp of the tooth and the disease, and who goes so far as to advocate the removal of the pulp of every tooth that has the disease.

*Dr. Kirk.*—I think there is no question whatever as to the intimate relation of the pulp to the peridental membrane, and when we consider that the tissues are all nourished by the blood-stream it is easy to understand that when the blood carries an irritant substance with it the effect may be so slight as to stimulate the building up of tissue resulting in hypercementosis, or it may induce a condition of hyperactivity of the pulp-tissues and produce pulp-nodules, for instance. There is a connection between the two, but I do not understand just why the destruction of the pulp would have any effect in curing the peridental membrane. I do not think that it would. It has been advocated by several, but I cannot conceive of any good scientific reason for the operation, except that the pulp may become a source of irritation itself; in that case it should be removed.

*Dr. L. W. Fowler.*—A gentleman came to me to have a tooth filled. I found that the pulp of the tooth was devitalized. I treated it. I also found that every other tooth was badly affected with pericementitis and loose to a high degree, with the exception of this tooth of which the pulp was dead. I would like to know, if the pulp had been dead for some time, with a filling over it, why it remained firm while the other teeth with live pulps were loosened?

*Dr. W. I. Brigham.*—Did the doctor ever notice that teeth that were subject to this disease were not subject to decay? In regard to nutrition, I have a little patient who is thirteen years old, a pupil in the high school, for whom I think I must have filled at least ten cavities within the last few days. It is a question whether that girl is not going beyond her strength. I think that the teeth show want of nutrition quicker than any other part of the body. I know a man for whom I filled some teeth several years ago, and within a year nearly every tooth he had was decayed. I did nothing for him, as he left town. Lack of nutrition, I think, was the cause.

*Dr. Kirk.*—There seems to be an idea that malnutrition will bring about the condition that will produce caries. But in a lowered vitality the enamel is not included, because we admit that it is not vital in the ordinary sense of that term. It must be what Dr. Black has contended for, and what Dr. Andrews believes to be true; it must be something in the environment of the enamel, in the fluids, or in the tissues of the mouth that is affected by these alterations in vital conditions, so that the mouth becomes a more suitable habitat for the germs of caries or for their production.

From what has been said as bearing upon the paper, I fear that in one or two points I may have misrepresented myself. I tell my students that pyorrhœa alveolaris is not a disease. I did not say that phagedenic pericementitis is not a disease. Pyorrhœa alveolaris is a flowing of pus from the alveolus; that is not a disease, but the symptom of a disease.

With regard to this question of education, which has been so pointedly stated by Dean Smith and has been alluded to by Dr. Clapp, I will say, for the encouragement of Dr. Piper, that he need not worry himself any longer with regard to his ignorance on these matters by comparison with the medical profession. The only circumstance that drove me to this study was that I could find no physician who knew anything about it. I went from one physician to another, and at last I have gotten a few interested in the subject, and I turn over these cases to them.

What is gout? You will find a great many varieties of gout. I look upon it as a condition of malnutrition, and in that sense a cause of diminished vitality. When we depress vitality low enough we have death, or, if you please, necrosis, which is another name for death. We may lower vital resistance to a point where we bring about disease, where we become the prey of bacteria. I said that I admit bacterial factor, but there is something preceding that and it is this destruction of our sources of internal resistance,—our standing army, so to speak, which leaves us a prey to the bacteria of disease. I think the suggestion made by Dr. Peirce carries a great deal of truth with it. I believe that the principle he has called attention to is the key to the solution of the problem of phagedenic pericementitis. And as to our friend who knows all about gout but did not have it, but had erosion, that seems to me to be an impossibility. Either he has erosion and gout, or else he does not know that he has a gouty diathesis. It can be determined by a microscopical and chemical examination of the blood. A great many people, when you ask them if they have gout, know nothing about it. You ask them if they have rheumatism and they say no. I have asked some, "Are you in good health?" and they said, "Yes"; but I found that they were not in good health; they did not realize that they had departed from normal health standards; but by putting them in a normally healthy condition it was easy to demonstrate to them that they had not been perfectly healthy before. I got at it negatively, and life was a new thing to them.

Marion L. Woodward, D.D.S.,
*Secretary Alumni Association, Boston Dental College.*

